# Roles of A-Kinase Anchoring Proteins and Phosphodiesterases in the Cardiovascular System

**DOI:** 10.3390/jcdd5010014

**Published:** 2018-02-20

**Authors:** Maria Ercu, Enno Klussmann

**Affiliations:** 1 Max Delbrück Center for Molecular Medicine Berlin (MDC), Berlin 13125, Germany; maria.ercu@mdc-berlin.de; 2 DZHK (German Centre for Cardiovascular Research), partner site Berlin 13347, Germany

**Keywords:** cAMP, compartmentalization, A-kinase anchoring proteins (AKAP), cyclic nucleotide phosphodiesterases (PDE), PDE inhibitors

## Abstract

A-kinase anchoring proteins (AKAPs) and cyclic nucleotide phosphodiesterases (PDEs) are essential enzymes in the cyclic adenosine 3′-5′ monophosphate (cAMP) signaling cascade. They establish local cAMP pools by controlling the intensity, duration and compartmentalization of cyclic nucleotide-dependent signaling. Various members of the AKAP and PDE families are expressed in the cardiovascular system and direct important processes maintaining homeostatic functioning of the heart and vasculature, e.g., the endothelial barrier function and excitation-contraction coupling. Dysregulation of AKAP and PDE function is associated with pathophysiological conditions in the cardiovascular system including heart failure, hypertension and atherosclerosis. A number of diseases, including autosomal dominant hypertension with brachydactyly (HTNB) and type I long-QT syndrome (LQT1), result from mutations in genes encoding for distinct members of the two classes of enzymes. This review provides an overview over the AKAPs and PDEs relevant for cAMP compartmentalization in the heart and vasculature and discusses their pathophysiological role as well as highlights the potential benefits of targeting these proteins and their protein-protein interactions for the treatment of cardiovascular diseases.

## 1. Introduction

Cardiovascular diseases (CVD) represent the leading cause of death worldwide and hypertension is the main risk factor for such conditions [1]. Treatments targeting the causes of cardiovascular diseases such as hypertension or heart failure are rare [2]. 

The second messenger cyclic adenosine 3′-5′ monophosphate (cAMP) is ubiquitous and functions as a signal transducer of many extracellular cues [3]. It regulates a variety of biological processes that are essential for, among others, proper cardiac function and it is involved in disease [4,5]. cAMP exerts its effects via activation of downstream effector proteins, i.e., cAMP-dependent protein kinase A (PKA), exchange proteins activated by cAMP (Epac) and cyclic nucleotide-gated ion channels (CNG), hyperpolarization-activated cyclic nucleotide-gated channels (HCN) and the recently identified Popeye domain containing (POPDC) proteins [6,7,8].

The plethora of extracellular signals, the limited number of intracellular cAMP effectors and the requirement of a specific biological response to each of the external signals imply a tight control of the intracellular signaling. This is achieved through signaling in defined cellular compartments. Local cAMP pools are established by the interplay of essentially four processes within the cell, namely cAMP synthesis, its diffusion, formation of multi-protein signaling complexes and cAMP degradation. In the heart, stimulation of β-adrenoceptors (β-ARs) triggers the activation the α-subunits of the stimulatory G proteins (G_s_), which in turn stimulate adenylyl cyclases (ACs) to convert ATP to cAMP [9]. The formation of multi-protein signaling complexes in the cAMP signaling pathway is orchestrated by the family of A-kinase anchoring proteins (AKAPs), which act as scaffolds and engage in direct protein-protein interactions, including with PKA, and target them to defined subcellular compartments [10,11,12,13,14,15]. AKAPs play essential roles both in the heart and vascular physiology by coordinating complexes involved in the regulation of various processes including endothelial-barrier function [16,17] cardiac contraction and relaxation [18,19,20,21] and action potential duration [22,23]. AKAPs apparently play a role in several pathophysiological conditions in the cardiovascular system, e.g., in the heart their dysregulation is associated with heart failure [24,25]. 

Termination of cAMP signaling is predominantly achieved by hydrolysis of the phosphodiester bond within the second messenger, a reaction catalyzed by cyclic nucleotide phosphodiesterases (PDEs) [26]. Various PDE families regulate different aspects of cardiac and vascular muscle functions [27], such as the endothelial barrier function [28,29], the Ca^2+^ handling and thus contractility [30] and the basal pacemaking activity [31]. PDEs are also involved in the pathological cardiac remodeling and dysfunction [4,32,33,34,35].

The aim of this review is to provide an overview over the AKAPs and PDEs that are relevant in the compartmentalization of cAMP signaling in the cardiovascular system, to discuss their role in physiology and pathophysiology and the potential of these proteins and their protein-protein interactions as pharmacological targets in cardiovascular diseases.

## 2. A-kinase Anchoring Proteins (AKAPs)

AKAPs are a family of over 40 different scaffolding proteins and are key players in the spatio-temporal control of cAMP-dependent signaling by targeting PKA and additional signaling proteins including ACs, PDEs, further protein kinases and phosphatases to specific subcellular compartments [11,36,37,38]. PKA is the major downstream effector of cAMP. It is a serine/threonine kinase with broad specificity that controls many cellular processes, e.g., metabolism, cell growth, cell division and cardiac myocyte contraction [39]. It is a heterotetramer that consists of two catalytic subunits (Cα, Cβ or Cγ) kept in an inactive state by two regulatory RI (RIα or RIβ) or RII (RIIα or RIIβ) subunits that are organized as homodimers in the holoenzyme [40]. Upon cAMP binding to the R subunits, the C subunits are released and thus activated and subsequently phosphorylate local substrates [39]. This view was recently confirmed by quantitative mass spectrometric analyses [41]. However, PKA holoenzyme can also be active, as indicated by early biochemical experiments [42,43]. This notion was supported by recent fluorescence resonance energy transfer (FRET) imaging-based experiments, which suggested that physiological cAMP levels promote only minimal dissociation of the C subunits from the holoenzyme, thereby limiting the range of PKA action to the substrates in the immediate proximity [44]. Thus, it appears that both PKA holoenzyme and/or the dissociated C subunits can be active.

**Figure 1:**
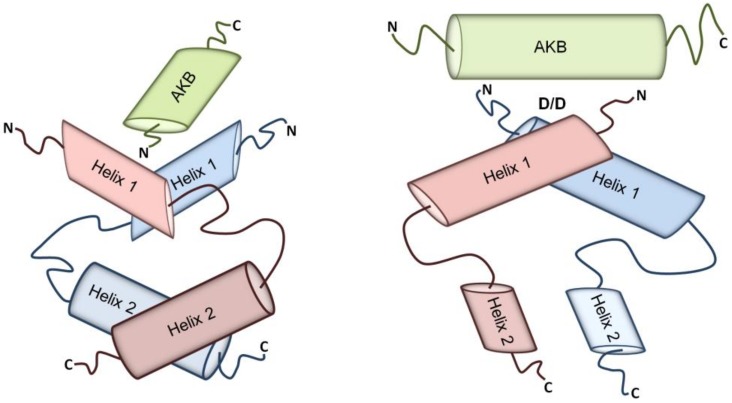
**Schematic representation of A-kinase anchoring proteins (AKAP)-protein kinase A (PKA) interactions displayed at two different angles.** The amphipathic AKB helix of AKAPs docks into the hydrophobic groove formed by the dimers of the N-terminal D/D domains of regulatory subunits of PKA. *AKB* A-kinase-binding domain; *D*/*D* dimerization and docking domain.

The structural feature that all AKAPs share is their ability to bind PKA via their A-kinase binding domains (AKBs), a structurally conserved amphipathic helix of 14–18 amino acids that docks into the hydrophobic groove formed by the N-terminal dimerization/docking (D/D) domains upon R subunits’ dimerization (Figure 1) [45,46,47,48]. Despite the fact that most AKAPs bind to PKA-RII subunits [37,49], there are the so-called dual specific AKAPs [50] that can bind both RI and RII subunits as well as AKAPs that specifically bind RI subunits (e.g., sphingosine kinase interacting protein (SKIP) and small membrane (sm) AKAP) [51,52,53]. Recently, hydrophilic anchor points have been identified within and outside the amphipathic helix forming the AKB that are involved in determining the affinity of the binding between an AKAP and the D/D domain. These observations suggest that targeting the amino acids that act as anchor points could lower the binding affinity or even prevent the interaction, making them candidates for pharmacological targeting. Moreover, targeting the anchor points makes the development of selective inhibitors of specific AKAP-PKA interactions feasible [48]. Selective inhibitory agents would be valuable tools for the investigation of cellular functions of individual AKAP-PKA interactions and could be starting points for drug development efforts.

### 2.1. AKAP Subcellular Localization 

Targeting of AKAPs to specific subcellular compartments is essential for a coordinated cAMP-dependent signaling response, including accurate PKA-catalyzed substrate phosphorylation [54]. AKAPs can be directed to various cellular compartments, including the plasma membrane (PM, e.g., AKAP18α, AKAP18β, AKAP79 [55,56,57]), the sarcoplasmic reticulum (SR, e.g., AKAP18δ [20]), the cytosol (e.g., SKIP, GSKIP [51,58,59,60,61]), the cytoskeleton (e.g., gravin, ezrin [62]), the mitochondria (e.g., D-AKAP1 [63]) and the nucleus (e.g., pericentrin and AKAP350 [64,65]).

### 2.2. AKAPs in the Cardiovascular System

Several AKAPs are expressed in the cardiovascular system (Table 1). They regulate a variety of processes and are key proteins in maintaining the homeostatic functioning of the heart and vasculature [66]. For instance, gravin and AKAP220 are involved in maintaining the vascular integrity [16,17]. Homeostasis of the vascular tone is achieved through tight control of the balance between contraction and relaxation of vascular smooth muscle cells (VSMC), processes in which AKAP79 is involved [67,68]. Ca^2+^ handling and thus cardiac myocyte contractility is regulated by several macromolecular protein complexes whose platforms are AKAPs, e.g., AKAP18α, γ and δ, mAKAPβ [19,20,21,69]. The AKAP Yotiao is the key player in cardiac myocyte repolarization that follows contraction [22]. Several AKAPs are involved in stress response-induced cardiac myocyte hypertrophy, including AKAP-Lbc and mAKAPβ [70,71]. AKAP79 and gravin are important for the recycling of β_1_-ARs and β_2_-ARs, respectively [72,73].

#### 2.2.1. AKAPs Regulating the Endothelial Barrier Function

The vascular endothelium lining the intima of blood vessels consists of a layer of endothelial cells tightly adherent to each other through cell-cell junctions. A healthy endothelium plays an essential role in the proper functioning of the vascular system. It regulates macromolecular permeability and anti-inflammatory, anti-thrombotic and anti-hypertrophic responses. Inflammatory conditions trigger pathological changes in the vascular system that lead to endothelial dysfunction, a state in which pathologically activated endothelial cells lose their barrier properties and initiate expression of pro-inflammatory adhesion molecules on their surface [74]. This results in increased vascular permeability allowing the infiltration of various molecules such as lipoproteins into the sub-endothelial space, and of circulating immune cells (e.g., monocytes). Ultimately, this leads to severe pathological conditions including atherosclerosis, allergy and sepsis [75,76]. 

AKAP-mediated PKA compartmentalization is essential for the maintenance of proper endothelial barrier function [16,17,77]. The vascular endothelium integrity is mainly dependent on tight junctions (TJs), important in sealing space between adjacent cells, and on adherens junctions, which assure direct contacts with the actin cytoskeleton of neighboring cells, thus providing mechanical strength. AKAP220 associates with PKA, β-catenin and the endothelial adherens junctions protein VE-cadherin, tethering PKA in close proximity to the cell-cell junctions [16]. Gravin (also known as AKAP12 or AKAP250) promotes vascular integrity by regulating the actin cytoskeleton via p21-activated kinase family proteins 2 (PAK2), an actin cytoskeletal regulator and afadin (AF6), a linker of the actin cytoskeleton with intercellular adhesion molecules [17]. Rac1 is a member of the Rho family of small GTPases, which upon activation strengthens the adherens junctions and the cortical actin skeleton, thereby preserving the endothelial barrier [78]. Simultaneous depletion of gravin and AKAP220 inhibited cAMP-mediated Rac1 activation, underlining the importance of these AKAPs in preventing endothelial dysfunction [16].

One other member of the AKAP family is involved in maintaining vascular integrity, the long isoform of AKAP9. Following Epac1 activation, AKAP9 contributes to microtubule growth regulation and is essential for preserving the endothelial barrier [79].

#### 2.2.2. AKAPs Regulating the Vascular Tone

Homeostasis of the vascular tone is maintained by a tight balance between dilation and constriction of blood vessel endothelium; the main regulator is the renin-angiotensin-aldosterone system (RAAS). The main effector molecule of this system is angiotensin II (AngII), which exerts most of its effects via angiotensin type I receptors (AT_1_R). For instance, arterial smooth muscle contraction is induced by AngII-dependent stimulation of AT_1_R, localized at the sarcolemma, and subsequent activation of phospholipase C (PLC), which catalyzes the hydrolysis of phosphatidylinositol 4,5-bisphosphate (PIP2) to diacylglycerol (DAG) and inositol 1,4,5-trisphosphate (IP3). DAG activates protein kinase C (PKC), which in turn phosphorylates L-type Ca^2+^ (Ca_V_1.2) channels, thereby increasing their open probability and increasing Ca^2+^ entry into the cytosol [75]. 

In arterial smooth muscle cells, the activity of a specific subpopulation of Ca_V_1.2 channels is regulated by AKAP79 (AKAP5/AKAP75/AKAP150)-dependent targeting of PKCα to the sarcolemma, which facilitates phosphorylation of the channels and increases their open probablility [80]. By affecting the opening probability of specific Ca_V_1.2 channels, the AKAP79 complex regulates the so-called “Ca_V_1.2 sparklets”, which refers to local elevations of intracellular Ca^2+^ pools that directly induce contraction of the VSMCs. The sparklets increase the vascular tone [67]. In addition, AKAP79 facilitates and most probably stabilizes the coupling of small clusters of adjacent Ca_V_1.2 channels, which can then open synchronously and generate large Ca_V_1.2 sparklets, thus increasing the contractile force [81,82]. Prolonged Ca_V_1.2 channel activity and thus persistent Ca_V_1.2 sparklets could lead to vascular dysfunction and eventually contribute to AngII-induced hypertension [80]. 

Transient receptor potential vanilloid 4 (TRPV4) channels are Ca^2+^ permeant channels that unlike the Ca_V_1.2 channels, promote relaxation upon activation. Both Ca_V_1.2- and TRPV4-mediated Ca^2+^ influxes activate adjacent ryanodine receptors (RyR), leading to release of Ca^2+^ from the SR into the cytosol in the form of Ca^2+^ sparks. While the Ca_V_1.2-mediated Ca^2+^ influx increases contraction, the local TRPV4-generated Ca^2+^ sparks activate the large-conductance, Ca^2+^-activated K^+^ (BK) channels, which promote membrane hyperpolarization and closure of the Ca_V_1.2 channels, ultimately resulting in relaxation [83,84]. In the arterial smooth muscle cells, AngII increased TRPV4 activity via PKC, which is tethered to the sarcolemma in close proximity of the channel by AKAP79, thus opposing the Ca_V_1.2 channel-induced vasoconstriction [68].

In conclusion, AKAP79 plays an essential role in the control of arterial vascular tone by regulating two opposing processes, contraction and relaxation of arterial myocytes.

#### 2.2.3. AKAPs Controlling Excitation-Contraction Coupling

The cycling of Ca^2+^ between the cytosol and the SR is at the basis of cardiac contraction and relaxation. Key players in these processes are L-type Ca^2+^ Ca_V_1.2 channels, RyR_2_, SR Ca^2+^ ATPase 2 (SERCA2) and the Na^+^/Ca^2+^ exchanger. More specifically, upon sarcolemma depolarization, Ca_V_1.2 channels located at the T tubules open allowing Ca^2+^ influx into the cardiac myocyte. This causes Ca^2+^-induced Ca^2+^ release from the SR into the cytosol through RyR_2_ located at the SR. The Ca^2+^, upon interaction with troponin T located on the thin myofibers, promotes contraction. Relaxation occurs via SERCA2-mediated Ca^2+^ re-uptake into the SR and through Ca^2+^ transport out of the cell by Na^+^/Ca^2+^ exchangers [85]. SERCA2 is activated upon the phosphorylation and subsequent dissociation of phospholamban (PLN), a SR phosphoprotein [20,86,87].

β-ARs introduce a further layer into the regulation of cardiac myocyte contractility. Their stimulation induces PKA-dependent phosphorylation of several proteins involved in Ca^2+^ handling, e.g., the Ca_V_1.2 channels, RyR_2_ and PLN. These phosphorylations are facilitated by distinct AKAPs.

AKAP18α is a membrane-associated scaffolding protein and is the smallest AKAP7 gene transcript, comprising 81 amino acids. AKAP18α promotes cardiac contractility by mediating the PKA-dependent phosphorylation of Ca_V_1.2 channels at Serine 1928 (Ser1928) on its α subunit and at multiple sites on its β subunit, which enhances the open probability of the channel and increases the Ca^2+^ current [69,88]. The activity of a subset of Ca_V_1.2 channels associated with caveolin-3 (Cav3) is regulated by PKA phosphorylation of the specific channel subpopulation mediated by an AKAP79 (AKAP5/AKAP75/AKAP150)-based macromolecular complex consisting of β-AR, PKA, AC5/6 and protein phosphatase calcineurin (PP2B) [89]. The muscle selective AKAP, mAKAPβ (a short version of mAKAP) associates with RyR_2_ at the SR and thereby facilitates the PKA phosphorylation of the channel, leading to enhanced opening of the channel and subsequent enhanced Ca^2+^ release from the SR into the cytosol [19]. In addition, mAKAPβ interacts with the Na^+^/Ca^2+^ exchanger 1 at the sarcolemma and promotes the PKA-dependent activation of the exchanger, resulting in increased Ca^2+^ efflux [90,91]. AKAP18δ (rat heart) and AKAP18γ (human heart) facilitate the PKA phosphorylation of PLN and promote its dissociation from SERCA2 and hence activation of the ATPase, thus enhancing the re-uptake of Ca^2+^ into the SR [20,21,92].

#### 2.2.4. AKAPs Regulating Cardiac Repolarization

The cardiac repolarization phase is initiated by the slow heart potassium current (I_Ks_) moving outwards through the I_Ks_ potassium channel, a macromolecular complex consisting of a pore-forming α subunit (KCNQ1) and a regulatory β subunit (KCNE1) along other intracellular proteins [93]. The AKAP Yotiao, the smallest transcript of the *AKAP9* gene, is essential for cardiac repolarization since it mediates the PKA-dependent phosphorylation of KCNQ1 and therefore regulates the activity of the I_Ks_ potassium channel [22]. Mutations in the KCNQ1 subunit or Yotiao increase the duration of the action potential and lead to type I long-QT syndrome (LQT1), a channelopathy that can elicit fatal arrhythmia [94]. Another AKAP that contributes to the regulation of cardiac action potentials is the dual specific D-AKAP2 (AKAP10). A single-nucleotide polymorphism (SNP) in its PKA binding domain causes a decrease in the PR interval in the electrocardiogram, which in turn can cause arrhythmias and sudden cardiac death [54,95,96,97]. 

#### 2.2.5. AKAPs Involved in Cardiac Stress Response

Cardiac hypertrophy is a stress-induced adaptation to maintain normal heart function [23,25]. At the cellular level, it is characterized by the upregulation of specific genes that promote the non-mitotic growth of cardiac myocytes [98]. AKAP-Lbc encodes in addition to its AKAP function for a guanine nucleotide exchange factor (GEF) that directly binds and activates the GTP-binding protein RhoA [99,100,101,102]. The interaction is involved in both cardiac development [103] and pathological cardiac myocyte hypertrophy [70]. α_1_-AR stimulation enhances the RhoGEF activity of AKAP-Lbc, which in turn activates RhoA, contributing to a pathological increase in the hypertrophic response [70]. PKA-mediated phosphorylation at Ser1565 of AKAP-Lbc leads to the recruitment of 14-3-3 proteins, which inhibit the Rho-GEF activity of the anchoring protein [104]. Also, an AKAP-Lbc-dependent signalosome mediates the activation and cytosolic release of activated protein kinase D (PKD), which has been shown to promote cardiac hypertrophy by facilitating the nuclear export of histone deacetylase 5 (HDAC5) [105,106].

Another AKAP that plays a central role in modulating stress signal-induced hypertrophic pathways is mAKAPβ. It coordinates a variety of cAMP-responsive enzymes. This anchoring protein is targeted to the nuclear envelope of cardiac myocytes via an interaction with nesprin-1α [107]. At the SR it can integrate and transduce a variety of hypertrophic signals [71]. For instance, mAKAPβ-mediated PKA phosphorylation and subsequent activation of RyR_2_ located at the nuclear envelope promotes the activation and nuclear translocation of the pro-hypertrophic transcription factor nuclear factor of activated T cells (NFAT) [108]. In addition, a mAKAPβ-based signalosome consisting of PKA, PDE4D3, Epac1, ERK5 and PP2A promotes ERK5-induced cardiomyocyte hypertrophy [71,109]. Cardiac remodeling can also be regulated by hypoxia, a process in which a mAKAP-based protein complex consisting of hypoxia-inducible factor 1α (HIF-1α), prolyl hydroxylase domain protein (PHD), the von Hippel-Lindau protein (pVHL) and the E3 ligase designated seven in absentia homolog 2 (Siah2) plays a role. More specifically, when oxygen levels are reduced, mAKAP promotes the degradation of PHD and thereby facilitates an increase in HIF-1α levels, which regulates transcription of genes that promote cell survival [110].

Other AKAPs that are thought to be involved in the cardiac stress response are D-AKAP1 and SKIP [111,112]. D-AKAP1 is a scaffolding protein of the outer mitochondrial membrane, which is protective against cardiac hypertrophy since its overexpression leads to cardiac myocyte cell size reduction and inhibition of the β-AR agonist isoproterenol-induced hypertrophy [111]. Moreover, D-AKAP1 expression maintains the mitochondrial structure and function in the heart and reduces the infarct size, cardiac remodeling and mortality under conditions of ischemia, i.e., after myocardial infarction [113]. 

SKIP plays an important role in the generation of the cardioprotective and anti-apoptotic lysophospholipid sphingosine-1-phosphate (S1P) produced upon myocardial ischemia-reperfusion injury [112]. It is involved in the regulation of sphingosine kinase type 1 (SPHK1), which upon activation phosphorylates sphingosine to form S1P [114].

#### 2.2.6. AKAPs Involved in the β-ARs Desensitization/Resensitization Cycle

Upon activation, β-ARs are phosphorylated and subsequently bind β-arrestin, which prevents further ligand binding leading to receptor desensitization. The phosphorylated β-ARs are internalized and reach the early endosomes where they undergo resensitization after PP2A-mediated dephosphorylation. Upon resensitization, the non-phosphorylated receptors are recycled to the plasma membrane where they can bind further ligands. Therefore, β-AR desensitization and resensitization are essential processes in maintaining the proper functioning of the receptor [115].

Gravin and AKAP79 are important in the desensitization/resensitization cycle [72,73]. A gravin-based complex consisting of PKA, PKC, PP2B, β-arrestin and G protein-linked receptor kinase 2 (GRK2) is essential for the desensitization and resensitization of the β_2_-ARs, with which it interacts at their C-terminal tail [72]. AKAP79 mediates the PKA-dependent phosphorylation of the β_1_-ARs by also binding to the C terminus of the receptor, leading to their recycling and resensitization [73].

### 2.3. Aberrant cAMP Compartmentalization Can be Visualized

Dysregulation of local cAMP signaling is associated with cardiovascular diseases, e.g., maladaptive cardiac remodeling and heart failure [116,117]. FRET-based imaging using genetically encoded sensors (cAMP-binding and PKA activity reporters) is utilized to visualize local cAMP signaling components and real-time changes in cAMP levels with high spatio-temporal resolution [118,119,120,121]. Such sensors can be targeted to various subcellular locations including in cardiac myocytes in close proximity of sarcolemmal ion channels and SR proteins involved in Ca^2+^ handling, e.g., RyR_2_ and SERCA2a [122,123,124,125]. In addition, the FRET-based reporters can be used for cAMP imaging in intact cardiac tissue as well as in ex vivo and in vivo hearts [126]. For monitoring activities of signaling molecules in their cognate microdomains, FRET approaches have also been combined with other techniques such as scanning ion-conductance microscopy (SICM), a non-optical method that allows the imaging of both cell membrane morphology and functional parameters at resolutions in the nanometer range [127,128,129,130]. 

## 3. Cyclic Nucleotide Phosphodiesterases (PDEs)

**Figure 2:**
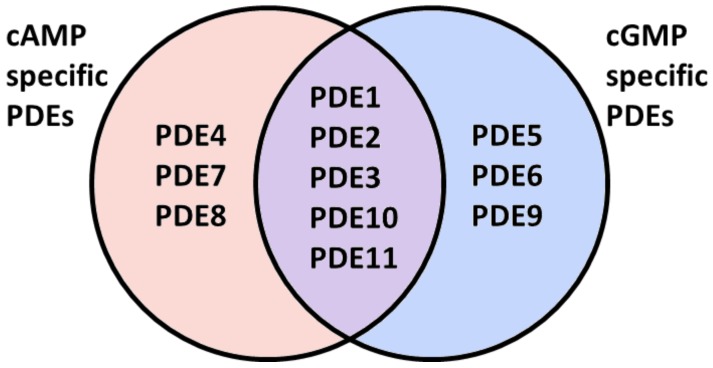
Substrate specificity of individual phosphodiesterase (PDE) families.

Hydrolysis by PDEs is the main route for lowering of intracellular levels of cAMP and cGMP and is essential for the spatio-temporal regulation of cyclic nucleotide-dependent signaling [26]. The PDE superfamily consists of 21 genes that give rise to more than 100 proteins due to differential transcription initiation sites and alternative splicing. PDEs are classified into 11 families (PDE1–PDE11). They differ in their primary structures, substrate specificities, mechanisms of regulation and kinetic properties [131]. Some PDE families selectively hydrolyze cAMP or cGMP, while others, the so-called dual specific PDEs, catalyze the hydrolysis of both second messengers (Figure 2) [132]. 

PDEs display a common general structure consisting of three components: a family-specific N-terminal regulatory domain, a conserved catalytic domain (25–52 % homology) and a C-terminal domain that can be either phosphorylated by the mitogen-activated protein kinase (MAPK) or prenylated [131,133,134,135]. The regulatory domains contain various structural features involved in the regulation (i.e., sites for covalent modifications, e.g., phosphorylation), binding of regulatory molecules (e.g., Ca^2+^-binding protein calmodulin), localization (targeting domains and protein-protein interaction motifs, e.g., AKAP-binding motifs) and dimerization (e.g., GAF domains) of the enzymes [131,136,137]. The catalytic domains feature in the active site a Zn^2+^ binding motif and an additional divalent metal binding site that is most probably occupied by Mg^2+^, but could also correspond to Mn^2+^ and Co^2+^ [138].

### 3.1. PDE Subcellular Localization

The subcellular localization of PDEs is key in achieving compartmentalized cyclic nucleotide signaling and, therefore, in the generation of specific physiological responses [136]. PDEs are located at various intracellular locations, e.g., the cytosol (e.g., PDE3A3 and PDE5 [139,140]), plasma membrane (e.g., PDE2A, PDE3A1, PDE6α, PDE6β [139,141,142]), the Golgi–centrosome (e.g., PDE7A1 [143]) and nuclear regions (e.g., PDE9A1, PDE9A16, PDE9A17 [144]). 

### 3.2. PDEs in the Cardiovascular System

Members from most PDE families are expressed in the cardiovascular system and regulate a variety of processes essential for the proper functioning of the heart and vasculature (Table 2). The PDE families 2, 3, 4 and 5, for example, regulate the endothelial barrier function and are, therefore, of utmost importance in maintaining vascular integrity [28,145,146]. Several members of the PDE families 2, 3, 4, 5 and 8 are involved in the control of cardiac contractility [30,120,147,148,149,150]. In addition, the basal pace-making activity of the sinoatrial (SA) node of the heart is regulated by two PDE families, namely PDE3 and PDE4 [151]. In addition, various PDE family members, PDE1A, PDE3A, PDE4B, PDE4D, PDE5 and PDE9A, are implicated in the cardiac stress response, which triggers pathological cardiac remodeling and ultimately cardiac dysfunction (e.g., heart failure and arrhythmias) [34,152,153,154,155,156].

#### 3.2.1. PDE3A and Autosomal Dominant Hypertension with Brachydactyly (HTNB)

**Figure 3:**
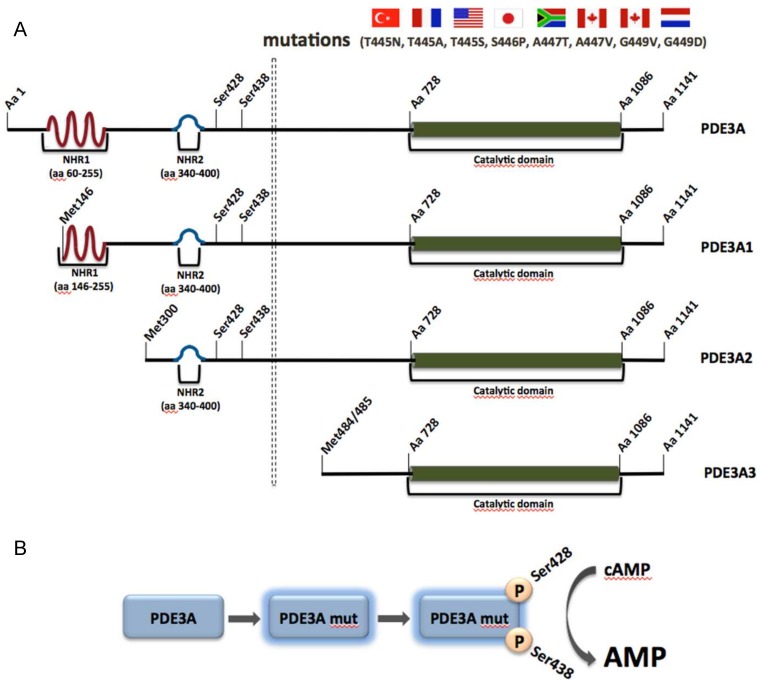
Schematic representation of the PDE3A gene, PDE3A protein isoforms and the hyperphosphorylation caused by the identified mutations. (**A**) Eight mutations have been identified in families from the countries indicated by the flags. The mutations cluster within a region of the gene encoding amino acids 445 and 449. The mutations cause hyperphosphorylations of Ser428 and Ser438. The N-terminal hydrophobic region (NHR) 1 of PDE3A1 comprises four transmembrane domains, while NHR2 contains no typical transmembrane region but a cluster of hydrophobic amino acids. PDE3A2 contains only NHR2 and PDE3A3 lacks all N-terminal hydrophobic regions (for details see text). (**B**) The hyperphosphorylation increases cyclic adenosine 3′-5′ monophosphate (cAMP) hydrolysis, causing low cAMP levels.

PDE3A along with PDE3B belongs to the PDE3 family, also known as the cGMP-inhibited cAMP PDE family, which is able to hydrolyze both cAMP and cGMP in a competitive manner. PDE3A is highly expressed and plays important roles in VSMCs, cardiac myocytes, platelets and oocytes, whereas PDE3B is mainly expressed in adipose and soft tissue. Upon alternative splicing, three PDE3A isoforms are generated, namely PDE3A1 (136 kDa), PDE3A2 (118 kDa) and PDE3A3 (94 kDa) (Figure 3A). They are located in different cellular compartments. PDE3A1 is the main isoform found in human cardiac myocytes and is predominantly located at membranes. It contains two N-terminal hydrophobic regions (NHR), of which the first one consists of four transmembrane domains. PDE3A2, which lacks the first but contains the second NHR can be both membrane-associated and cytosolic and is the main variant found in VSMCs. PDE3A3 is found only in the cytosol, since it lacks both previously mentioned hydrophobic regions. All three isoforms possess the same catalytic region and present high similarities regarding their catalytic activity and inhibitor sensitivity (Figure 3A) [157,158].

Mutations in genes encoding for distinct PDE family members can have detrimental effects and cause specific human diseases. One such example is represented by the Mendelian syndrome Autosomal-dominant hypertension with brachydactyly type E (HTNB), caused by missense mutations in the gene encoding for PDE3A [159]. The syndrome is characterized by an age-dependent progressive hypertension, brachydactyly type E and blood vessel hyperplasia [159]. If untreated, blood pressure increases by 50 mm Hg and patients die from stroke before age 50 years. Surprisingly, hypertension-associated end organ damage such as cardiac hypertrophy, kidney damage or hypertensive retinopathy is low [160,161].

Eight mutations in *PDE3A* were discovered in eight unrelated families from Turkey, France, the United States, South Africa, Canada, Netherlands and Japan. All these mutations were missense, gain of function mutations and found in close proximity to each other. The identified mutations cause amino acid substitutions in a region between amino acids 445 and 449 and increases of PKA-mediated phosphorylation of serine residues 428 and 438 of PDE3A1 and PDE3A2. The region is not present in PDE3A3 (Figure 3A) [159]. The substitutions lead to increased cAMP affinity and hydrolytic activity of the enzymes (Figure 3B). In addition, the hyperactive enzyme is erroneously localized in microsomal fractions from HeLa cells, suggesting that aberrant compartmentalization is detrimental in the cardiovascular system [159,161]. VSMCs from patients expressing the hyperactive version of the enzyme with the T445N substitution display higher proliferation rates, explaining the vascular phenotype. 

In the human heart, PKA-mediated phosphorylation of PDE3A1 induces its recruitment to an AKAP18-based signalosome in the heart that controls the Ca^2+^ reuptake into the SR and thereby participates in the control of cardiac relaxation [21]. An extended overview over functions of PDE3 in the heart was provided in recent reviews (e.g., [162,163]).

### 3.3. PDE Inhibitors

Due to their essential physiological and pathological roles in cyclic nucleotide signaling, PDEs are considered pharmacological targets for a variety of cardiovascular diseases, including atherosclerosis, hypertension, heart failure and intermittent claudication [26,164,165,166]. Several inhibitors of PDE3, 4 and 5 are approved as drugs, some of which are used for the treatment of cardiovascular diseases.

#### 3.3.1. PDE3

The PDE3 inhibitor cilostazol is an antiplatelet agent with vasodilatory and antiproliferative properties. It has been widely studied in a number of cardiovascular diseases including coronary and peripheral artery diseases and cerebrovascular disease [167]. Cilostazol is administered for the treatment of peripheral arterial circulatory disorders and also used as an antiplatelet agent in patients that underwent carotid artery stenting [168,169]. In addition, it is also approved for the treatment of intermittent claudication-induced symptoms [170,171,172]. Cilostazol appears to be a promising therapeutic agent for secondary prevention of stroke and was shown to improve right ventricular systolic function as well as to decrease pulmonary artery pressure [167,173]. PDE3 inhibitors inhibit neointima formation in a rat balloon double-injury model displaying neither cytotoxicity nor effects on VSMC migration, and thus are considered targets in preventing acute re-occlusion after angioplasties, e.g., percutaneous transluminal coronary angioplasty (PTCA) [174].

Milrinone is another PDE3 inhibitor. It has inotropic and vasodilatory properties, and is widely used in patients with end-stage heart failure in order to temporarily improve cardiac contractility (positive inotropic effect) and decrease vascular resistance. Taking into account that long-term administration of milrinone can induce apoptosis of cardiac myocytes, cardiac arrhythmias, hypotension and increases cardiovascular mortality, it is only used in a selected group of patients [170,175,176,177]. A potential explanation for the long-term PDE3 inhibitor therapy-induced mortality could be the fact that PDE3A inhibition induces cardiac myocyte apoptosis via a PDE3A-inducible cAMP early repressor (ICER) feedback loop. More specifically, PDE3A inhibition leads to PKA activation and ICER protein stabilization, which, in turn, promotes cardiac myocyte apoptosis. Therefore, therapeutic strategies that would diminish PDE3A activity without affecting the PDE3A-ICER feedback loop could promote the beneficial effects while by-passing the side effects [152,162]. Current research aims at determining the effects of milrinone on pulmonary hypertension and right ventricular failure, where it is believed to be particularly helpful [176]. 

#### 3.3.2. PDE4

The PDE4 family is encoded by four genes, *PDE4A*, *PDE4B*, *PDE4C* and *PDE4D* [178] and was shown to be involved in the excitation-contraction coupling regulation, especially in rodents. It has been recently suggested that PDE4 inhibitors could be beneficial in treating sepsis in infants with cardio-renal syndrome (CRS) since they are effective in improving cardiac function in a rat model suffering from sepsis-induced acute cardiac dysfunction and kidney injury [179]. In addition, PDE4 depletion stabilized the endothelial barrier by reducing the atrial natriuretic peptide (ANP)-induced vascular permeability and, therefore, was efficient in maintaining the plasma volume [180]. Despite the fact that PDE4A, PDE4B and PDE4D are expressed in the human and rodent heart, with PDE4D being the predominant isoform found in the human heart [181], there is no approval for a PDE4 inhibitor for the treatment of cardiovascular diseases. A highly selective PDE4 inhibitor, roflumilast, has been approved in various countries for the treatment of chronic obstructive pulmonary disease (COPD), a chronic inflammatory lung disease characterized by heavily breathing due to obstructive airflow from the lungs as well as a decline of lung function over time [182,183,184]. Another inhibitor, apremilast is employed for the treatment of psoriasis [185].

#### 3.3.3. PDE5

*PDE5A* is the sole gene coding for the PDE5 family, which plays an essential role in the cardiovascular system. PDE5 expression is low in the healthy cardiac tissue, whereas it is upregulated in the diseased heart [155,186]. PDE5 inhibition counteracts cardiac remodeling and fibrosis of isolated cardiac fibroblasts via repression of transforming growth factor (TGF)-β1-induced Smad signaling [187]. PDE5 depletion inhibits left ventricular remodeling induced by hypertrophic and pro-fibrotic stimuli [188]. Reduction in PDE5 expression was beneficial for chronic heart failure patients by enhancing the endothelium-dependent, flow-mediated vasodilation [189]. In addition, high PDE5 expression was identified in the hypertrophic human right ventricle and its inhibition enhanced contractility, particularly important for pulmonary hypertension [186]. PDE5 inhibitors such as sildenafil, vardenafil and tadalafil are approved for the treatment of erectile dysfunction and pulmonary hypertension but are not yet approved for the treatment of other cardiovascular diseases [26]. Nevertheless, recent studies suggest potential therapeutic benefits for PDE5 inhibitors, i.e., sildenafil and tadalafil in the treatment of myocardial infarction, ischemia/reperfusion injury, endothelial dysfunction, cardiac hypertrophy and heart failure [190,191].

#### 3.3.4. Potential for PDE1, PDE2, PDE8 and PDE9 Inhibitors

PDE1, PDE2, PDE8 and PDE9 inhibition is considered a therapeutic opportunity for the treatment of cardiovascular diseases but inhibitors are not approved.

The PDE1 family is encoded by three distinct genes, *PDE1A*, *PDE1B* and *PDE1C*, and is the only PDE family activated by calcium/calmodulin (Ca^2+^/CaM) binding [192,193]. Due to their potential to dilate coronary arteries, inhibition of PDE1 enzymes may be beneficial for the treatment of coronary artery disease (CAD) and angina pectoris [194]. Nuclear PDE1A is important for the proliferation of VSMCs and, therefore, could contribute to neointima formation in diseases, e.g., atherosclerosis and restenosis [195]. Thus, diminishing its expression could decrease pathological neointima development. In addition, inhibition of the PDE1 family might improve cardiopathy and pulmonary arterial hypertension since it decreases the structural remodeling process underlying these two conditions [196].

*PDE2A* is the only gene coding for the PDE2 family and plays a central role in the cardiac Ca_V_1.2 current regulation. The expression is up-regulated in human failing hearts [197,198]. Inhibition of PDE2 had a positive inotropic effect in dogs and mice, whereas its overexpression decreased the heart rate in mice. Interestingly, in a heart-specific PDE2-transgenic mouse model, increased PDE2 abundance prevents ventricular arrhythmias by inhibiting Ca^2+^ leak from the SR and helps in maintaining the contractile function of the heart after myocardial infarction [199]. On the contrary, a recent study in patients that had experienced an acute myocardial infarction (AMI) suggests that inhibition of endothelial PDE2A could have a beneficial effect and improve the clinical outcome. Hypoxia and pro-inflammatory cytokines such as tumor necrosis factor-α (TNF-α) promote PDE2A activation, which results in diminished submembrane cAMP levels and endothelial barrier disruption. This facilitates the extravasation of activated neutrophils and leads to inflammation in the early post-myocardial infarction phase [29].

The PDE8 family comprises two members, PDE8A and PDE8B and regulates excitation-contraction coupling in ventricular myocytes. More specifically, it has been suggested that PDE8A controls at least one cAMP pool involved in the cardiac myocyte-dependent Ca^2+^ cycling regulation. It was also observed that PDE8A deletion caused both increased RyR_2_ leak as well as enhanced Ca^2+^ refilling of the SR [150].

The PDE9 family is encoded by a single gene, *PDE9A*, and consists of more than 20 different splice variants. PDE9A expression was identified in human and rodent hearts, where its expression increased upon hypertrophy and heart failure development [156,200]. PDE9A depletion had a protective effect for the heart against pathological remodeling caused by pressure overload and it reversed a previously established heart disease without requiring the activity of NO synthase [156].

## 4. Concluding Remarks

Compartmentalized cyclic nucleotide signaling is found at the basis of precision of cellular signaling and its dysregulation is associated with various pathological conditions including several cardiovascular diseases. Local pools of cAMP are established by the interplay of cAMP synthesis, diffusion, degradation as well as positioning of the relevant signaling proteins. AKAPs and PDEs are essential players in these processes since they orchestrate the formation of multi-protein signaling complexes and terminate local cAMP signaling, respectively. This interplay ensures the spatio-temporal regulation of cyclic nucleotide-dependent signaling. Despite the fact that both molecules are key elements in the cAMP signaling pathway, very little is known with respect to their direct interaction or their interplay in the cardiovascular system. However, a few PDE-containing AKAP complexes have been identified; examples are the SERCA2/AKAP18 signalosome, which incorporates PDE3A1 upon its phosphorylation and is important for cardiac contractility, and the PDE4D3 containing mAKAPβ-based signalosome involved in cardiomyocyte hypertrophy regulation ([21,71,109]). Alterations in AKAP expression and their protein-protein interactions are associated with various cardiovascular diseases [12,24]. Hence the development of pharmacological agents targeting such dysregulated signaling components for evaluating their relevance as pharmacological targets is needed. First examples show that targeting AKAPs and their protein-protein interactions with small molecules is possible. For instance, an AKAP-PKA interaction inhibitor, FMP-API-1 [201] was identified. Recently, a novel small molecule, Scaff10-8, was developed, which inhibits the interaction of AKAP-Lbc and RhoA and prevents the AKAP-Lbc-mediated RhoA activation, an event pathologically activated in models of cardiac hypertrophy [102]. Further molecules directed against the AKAP-Lbc-RhoA interface have recently been identified and may serve to guide to further preclinical drug development efforts [202,203].

Approved inhibitors of PDEs target the catalytic activities of PDEs. However, the catalytic domains of the various members of individual families are identical and inhibition of one inhibits all. This lack of selectivity presumably explains PDE inhibitor therapy-associated side effects, which are frequent and dramatic over long-term administration [158]. PDE isoform-selective inhibition may be achieved through disruption of specific protein-protein interactions. and therefore the displacement of particular PDE isoforms from their subcellular compartments [204]. 

In conclusion, targeting proteins directing compartmentalized cAMP signaling, in particular AKAPs and PDEs, not only serves to understanding their role in heart and vascular physiology and pathophysiology but also has therapeutic potential for the treatment of a wide range of cardiovascular diseases.

## Figures and Tables

**Table 1:** Overview of AKAPs expressed in the heart and vasculature and of the cardiovascular processes that they regulate.

Common Name	Gene Name	Alternative Name	Regulated Cardiovascular Process
D-AKAP1	*AKAP1*	AKAP121/ AKAP149/AKAP84	Cardiac stress response
D-AKAP2	*AKAP10*	-	Cardiac repolarization
AKAP9 (long isoform)	*AKAP9*	-	Endothelial barrier function
AKAP18α AKAP18γ AKAP18δ	*AKAP7*	-	Excitation-contraction coupling
AKAP79	*AKAP5*	AKAP75/AKAP150	Vascular tone; Excitation-contraction coupling; β-AR desensitization/resensitization cycle
AKAP220	*AKAP11*	-	Endothelial barrier function
AKAP-Lbc	*AKAP13*	Brx-1/Proto-Lbc/ Ht31	Cardiac stress response
mAKAPβ	*AKAP6*	AKAP100	Excitation-contraction coupling; Cardiac stress response
AKAP Yotiao	*AKAP9*	GC-NAP	Cardiac repolarization
Gravin	*AKAP12*	AKAP250	Endothelial barrier function; β-AR desensitization/resensitization cycle
SKIP	*SPHKAP*	-	Cardiac stress response

**Table 2:** Overview of the PDE families expressed in the cardiovascular system and the corresponding cardiovascular processes that they regulate.

PDE Family	PDE Gene	Substrate Specificity	Regulated Cardiovascular Process
PDE1	PDE1A	cAMP, cGMP	Cardiac stress response
	PDE1B		
	PDE1C		
PDE2	PDE2A	cAMP, cGMP	Endothelial barrier function; Excitation-contraction coupling
PDE3	PDE3A	cAMP, cGMP	Endothelial barrier function; Excitation-contraction coupling; Basal pacemaking activity of the SA node; Cardiac stress response
	PDE3B		
PDE4	PDE4A	cAMP	Endothelial barrier function; Excitation-contraction coupling; Basal pacemaking activity of the SA node; Cardiac stress response
	PDE4B		
	PDE4C		
	PDE4D		
PDE5	PDE5A	cGMP	Endothelial barrier function; Excitation-contraction coupling; Cardiac stress response
PDE8	PDE8A	cAMP	Excitation-contraction coupling
	PDE8B		
PDE9	PDE9A	cGMP	Cardiac stress response

## References

[1] Lawes C.M., Vander Hoorn S., Rodgers A., Hypertension I.S.o. (2008). Global burden of blood-pressure-related disease, 2001. Lancet.

[2] Bolívar J.J. (2013). Essential hypertension: An approach to its etiology and neurogenic pathophysiology. Int. J. Hypertens..

[3] Beavo J.A., Brunton L.L. (2002). Cyclic nucleotide research—Still expanding after half a century. Nat. Rev. Mol. Cell Biol..

[4] Fischmeister R., Castro L.R., Abi-Gerges A., Rochais F., Jurevicius J., Leroy J., Vandecasteele G. (2006). Compartmentation of cyclic nucleotide signaling in the heart: The role of cyclic nucleotide phosphodiesterases. Circ. Res..

[5] Perera R.K., Nikolaev V.O. (2013). Compartmentation of cAMP signalling in cardiomyocytes in health and disease. Acta Physiol (Oxf).

[6] Lorenz R., Bertinetti D., Herberg F.W. (2017). cAMP-dependent protein kinase and cGMP-dependent protein kinase as cyclic nucleotide effectors. Handb. Exp. Pharmacol..

[7] Lezoualc’h F., Fazal L., Laudette M., Conte C. (2016). Cyclic AMP sensor epac proteins and their role in cardiovascular function and disease. Circ. Res..

[8] Brand T., Schindler R. (2017). New kids on the block: The popeye domain containing (popdc) protein family acting as a novel class of cAMP effector proteins in striated muscle. Cell Signal..

[9] Pierce K.L., Premont R.T., Lefkowitz R.J. (2002). Seven-transmembrane receptors. Nat. Rev. Mol. Cell Biol..

[10] Szaszák M., Christian F., Rosenthal W., Klussmann E. (2008). Compartmentalized cAMP signalling in regulated exocytic processes in non-neuronal cells. Cell Signal..

[11] Skroblin P., Grossmann S., Schafer G., Rosenthal W., Klussmann E. (2010). Mechanisms of protein kinase A anchoring. Int Rev. Cell Mol. Biol.

[12] Dema A., Perets E., Schulz M.S., Deák V.A., Klussmann E. (2015). Pharmacological targeting of AKAP-directed compartmentalized cAMP signalling. Cell Signal..

[13] Pidoux G., Taskén K. (2010). Specificity and spatial dynamics of protein kinase A signaling organized by A-kinase-anchoring proteins. J. Mol. Endocrinol..

[14] Scott J.D., Dessauer C.W., Taskén K. (2013). Creating order from chaos: Cellular regulation by kinase anchoring. Annu Rev. Pharmacol Toxicol.

[15] Nikolaev V.O., Zaccolo M. (2017). Microdomains in the Cardiovascular System.

[16] Radeva M.Y., Kugelmann D., Spindler V., Waschke J. (2014). PKA compartmentalization via AKAP220 and AKAP12 contributes to endothelial barrier regulation. PLoS One.

[17] Kwon H.B., Choi Y.K., Lim J.J., Kwon S.H., Her S., Kim H.J., Lim K.J., Ahn J.C., Kim Y.M., Bae M.K. (2012). AKAP12 regulates vascular integrity in zebrafish. Exp. Mol. Med..

[18] Gray P.C., Johnson B.D., Westenbroek R.E., Hays L.G., Yates J.R., Scheuer T., Catterall W.A., Murphy B.J. (1998). Primary structure and function of an A kinase anchoring protein associated with calcium channels. Neuron.

[19] Marx S.O., Reiken S., Hisamatsu Y., Jayaraman T., Burkhoff D., Rosemblit N., Marks A.R. (2000). Pka phosphorylation dissociates fkbp12.6 from the calcium release channel (ryanodine receptor): Defective regulation in failing hearts. Cell.

[20] Lygren B., Carlson C.R., Santamaria K., Lissandron V., McSorley T., Litzenberg J., Lorenz D., Wiesner B., Rosenthal W., Zaccolo M. (2007). AKAP complex regulates Ca^2+^ re-uptake into heart sarcoplasmic reticulum. EMBO Rep..

[21] Ahmad F., Shen W., Vandeput F., Szabo-Fresnais N., Krall J., Degerman E., Goetz F., Klussmann E., Movsesian M., Manganiello V. (2015). Regulation of sarcoplasmic reticulum Ca^2+^ atpase 2 (serca2) activity by phosphodiesterase 3a (pde3a) in human myocardium: Phosphorylation-dependent interaction of pde3a1 with serca2. J. Biol. Chem..

[22] Marx S.O., Kurokawa J., Reiken S., Motoike H., D’Armiento J., Marks A.R., Kass R.S. (2002). Requirement of a macromolecular signaling complex for beta adrenergic receptor modulation of the kcnq1-kcne1 potassium channel. Science.

[23] Frey N., Katus H.A., Olson E.N., Hill J.A. (2004). Hypertrophy of the heart: A new therapeutic target?. Circulation.

[24] Deák V.A., Klussmann E. (2016). Pharmacological interference with protein-protein interactions of akinase anchoring proteins as a strategy for the treatment of disease. Curr. Drug Targets.

[25] Diviani D., Reggi E., Arambasic M., Caso S., Maric D. (2016). Emerging roles of A-kinase anchoring proteins in cardiovascular pathophysiology. Biochim. Biophys. Acta.

[26] Maurice D.H., Ke H., Ahmad F., Wang Y., Chung J., Manganiello V.C. (2014). Advances in targeting cyclic nucleotide phosphodiesterases. Nat. Rev. Drug Discov..

[27] Kim G.E., Kass D.A. (2017). Cardiac phosphodiesterases and their modulation for treating heart disease. Handb. Exp. Pharmacol..

[28] Surapisitchat J., Jeon K.I., Yan C., Beavo J.A. (2007). Differential regulation of endothelial cell permeability by cgmp via phosphodiesterases 2 and 3. Circ. Res..

[29] Chen W., Spitzl A., Mathes D., Nikolaev V.O., Werner F., Weirather J., Špiranec K., Röck K., Fischer J.W., Kämmerer U. (2016). Endothelial actions of anp enhance myocardial inflammatory infiltration in the early phase after acute infarction. Circ. Res..

[30] Yan C., Miller C.L., Abe J. (2007). Regulation of phosphodiesterase 3 and inducible cAMP early repressor in the heart. Circ. Res..

[31] Galindo-Tovar A., Vargas M.L., Kaumann A.J. (2009). Phosphodiesterases PDE3 and PDE4 jointly control the inotropic effects but not chronotropic effects of (-)-cgp12177 despite PDE4-evoked sinoatrial bradycardia in rat atrium. Naunyn-Schmiedeberg’s Arch. Pharmacol..

[32] Ding B., Abe J.I., Wei H., Huang Q., Walsh R.A., Molina C.A., Zhao A., Sadoshima J., Blaxall B.C., Berk B.C. (2005). Functional role of phosphodiesterase 3 in cardiomyocyte apoptosis: Implication in heart failure. Circulation.

[33] Abi-Gerges A., Richter W., Lefebvre F., Mateo P., Varin A., Heymes C., Samuel J.L., Lugnier C., Conti M., Fischmeister R. (2009). Decreased expression and activity of cAMP phosphodiesterases in cardiac hypertrophy and its impact on beta-adrenergic cAMP signals. Circ. Res..

[34] Miller C.L., Oikawa M., Cai Y., Wojtovich A.P., Nagel D.J., Xu X., Xu H., Florio V., Rybalkin S.D., Beavo J.A. (2009). Role of Ca^2+^/calmodulin-stimulated cyclic nucleotide phosphodiesterase 1 in mediating cardiomyocyte hypertrophy. Circ. Res..

[35] Bobin P., Belacel-Ouari M., Bedioune I., Zhang L., Leroy J., Leblais V., Fischmeister R., Vandecasteele G. (2016). Cyclic nucleotide phosphodiesterases in heart and vessels: A therapeutic perspective. Arch. Cardiovasc. Dis..

[36] Rababa’h A., Singh S., Suryavanshi S.V., Altarabsheh S.E., Deo S.V., McConnell B.K. (2014). Compartmentalization role of A-kinase anchoring proteins (AKAPs) in mediating protein kinase A (PKA) signaling and cardiomyocyte hypertrophy. Int. J. Mol. Sci..

[37] Welch E.J., Jones B.W., Scott J.D. (2010). Networking with AKAPs: Context-dependent regulation of anchored enzymes. Mol. Interv..

[38] Langeberg L.K., Scott J.D. (2015). Signalling scaffolds and local organization of cellular behaviour. Nat. Rev. Mol. Cell Biol..

[39] Francis S.H., Corbin J.D. (1994). Structure and function of cyclic nucleotide-dependent protein kinases. Annu. Rev. Physiol..

[40] Taylor S.S., Ilouz R., Zhang P., Kornev A.P. (2012). Assembly of allosteric macromolecular switches: Lessons from PKA. Nat. Rev. Mol. Cell Biol..

[41] Walker-Gray R., Stengel F., Gold M.G. (2017). Mechanisms for restraining cAMP-dependent protein kinase revealed by subunit quantitation and cross-linking approaches. Proc. Natl. Acad. Sci. USA.

[42] Yang S., Fletcher W.H., Johnson D.A. (1995). Regulation of cAMP-dependent protein kinase: Enzyme activation without dissociation. Biochemistry.

[43] Kopperud R., Christensen A.E., Kjarland E., Viste K., Kleivdal H., Doskeland S.O. (2002). Formation of inactive cAMP-saturated holoenzyme of cAMP-dependent protein kinase under physiological conditions. J. Biol. Chem..

[44] Smith F.D., Esseltine J.L., Nygren P.J., Veesler D., Byrne D.P., Vonderach M., Strashnov I., Eyers C.E., Eyers P.A., Langeberg L.K. (2017). Local protein kinase A action proceeds through intact holoenzymes. Science.

[45] Ruehr M.L., Zakhary D.R., Damron D.S., Bond M. (1999). Cyclic amp-dependent protein kinase binding to A-kinase anchoring proteins in living cells by fluorescence resonance energy transfer of green fluorescent protein fusion proteins. J. Biol. Chem..

[46] Gold M.G., Lygren B., Dokurno P., Hoshi N., McConnachie G., Tasken K., Carlson C.R., Scott J.D., Barford D. (2006). Molecular basis of akap specificity for PKA regulatory subunits. Mol. Cell.

[47] Kinderman F.S., Kim C., von Daake S., Ma Y., Pham B.Q., Spraggon G., Xuong N.H., Jennings P.A., Taylor S.S. (2006). A dynamic mechanism for akap binding to rii isoforms of cAMP-dependent protein kinase. Mol. Cell.

[48] Götz F., Roske Y., Schulz M.S., Autenrieth K., Bertinetti D., Faelber K., Zühlke K., Kreuchwig A., Kennedy E.J., Krause G. (2016). AKAP18:Pka-riiα structure reveals crucial anchor points for recognition of regulatory subunits of PKA. Biochem. J..

[49] McSorley T., Stefan E., Henn V., Wiesner B., Baillie G.S., Houslay M.D., Rosenthal W., Klussmann E. (2006). Spatial organisation of AKAP18 and PDE4 isoforms in renal collecting duct principal cells. Eur. J. Cell. Biol..

[50] Huang L.J., Durick K., Weiner J.A., Chun J., Taylor S.S. (1997). Identification of a novel protein kinase A anchoring protein that binds both type i and type ii regulatory subunits. J. Biol. Chem..

[51] Kovanich D., van der Heyden M.A., Aye T.T., van Veen T.A., Heck A.J., Scholten A. (2010). Sphingosine kinase interacting protein is an A-kinase anchoring protein specific for type i cAMP-dependent protein kinase. Chembiochem.

[52] Means C.K., Lygren B., Langeberg L.K., Jain A., Dixon R.E., Vega A.L., Gold M.G., Petrosyan S., Taylor S.S., Murphy A.N. (2011). An entirely specific type i a-kinase anchoring protein that can sequester two molecules of protein kinase A at mitochondria. Proc. Natl. Acad. Sci. USA.

[53] Burgers P.P., Ma Y., Margarucci L., Mackey M., van der Heyden M.A., Ellisman M., Scholten A., Taylor S.S., Heck A.J. (2012). A small novel A-kinase anchoring protein (AKAP) that localizes specifically protein kinase A-regulatory subunit i (PKA-ri) to the plasma membrane. J. Biol. Chem..

[54] Tröger J., Moutty M.C., Skroblin P., Klussmann E. (2012). A-kinase anchoring proteins as potential drug targets. Br. J. Pharmacol..

[55] Fraser I.D., Tavalin S.J., Lester L.B., Langeberg L.K., Westphal A.M., Dean R.A., Marrion N.V., Scott J.D. (1998). A novel lipid-anchored A-kinase anchoring protein facilitates cAMP-responsive membrane events. EMBO J..

[56] Trotter K.W., Fraser I.D., Scott G.K., Stutts M.J., Scott J.D., Milgram S.L. (1999). Alternative splicing regulates the subcellular localization of A-kinase anchoring protein 18 isoforms. J. Cell Biol..

[57] Dell’Acqua M.L., Faux M.C., Thorburn J., Thorburn A., Scott J.D. (1998). Membrane-targeting sequences on AKAP79 bind phosphatidylinositol-4,5-bisphosphate. EMBO J..

[58] Hundsrucker C., Skroblin P., Christian F., Zenn H.M., Popara V., Joshi M., Eichhorst J., Wiesner B., Herberg F.W., Reif B. (2010). Glycogen synthase kinase 3β interaction protein functions as an A-kinase anchoring protein. J. Biol. Chem..

[59] Deák V.A., Skroblin P., Dittmayer C., Knobeloch K.P., Bachmann S., Klussmann E. (2016). The A-kinase anchoring protein gskip regulates gsk3β activity and controls palatal shelf fusion in mice. J. Biol. Chem..

[60] Dema A., Schröter M.F., Perets E., Skroblin P., Moutty M.C., Deàk V.A., Birchmeier W., Klussmann E. (2016). The A-kinase anchoring protein (AKAP) glycogen synthase kinase 3β interaction protein (gskip) regulates β-catenin through its interactions with both protein kinase a (PKA) and gsk3β. J. Biol. Chem..

[61] Scholten A., Poh M.K., van Veen T.A., van Breukelen B., Vos M.A., Heck A.J. (2006). Analysis of the cGMP/cAMP interactome using a chemical proteomics approach in mammalian heart tissue validates sphingosine kinase type 1-interacting protein as a genuine and highly abundant AKAP. J. Proteom. Res..

[62] Taskén K., Aandahl E.M. (2004). Localized effects of cAMP mediated by distinct routes of protein kinase a. Physiol. Rev..

[63] Huang L.J., Wang L., Ma Y., Durick K., Perkins G., Deerinck T.J., Ellisman M.H., Taylor S.S. (1999). Nh2-terminal targeting motifs direct dual specificity A-kinase-anchoring protein 1 (d-AKAP1) to either mitochondria or endoplasmic reticulum. J. Cell. Biol.

[64] Diviani D., Langeberg L.K., Doxsey S.J., Scott J.D. (2000). Pericentrin anchors protein kinase a at the centrosome through a newly identified rii-binding domain. Curr. Biol..

[65] Gillingham A.K., Munro S. (2000). The pact domain, a conserved centrosomal targeting motif in the coiled-coil proteins AKAP450 and pericentrin. EMBO Rep..

[66] Scott J.D., Santana L.F. (2010). A-kinase anchoring proteins: Getting to the heart of the matter. Circulation.

[67] Navedo M.F., Santana L.F. (2013). Cav1.2 sparklets in heart and vascular smooth muscle. J. Mol. Cell. Cardiol..

[68] Mercado J., Baylie R., Navedo M.F., Yuan C., Scott J.D., Nelson M.T., Brayden J.E., Santana L.F. (2014). Local control of trpv4 channels by AKAP150-targeted PKC in arterial smooth muscle. J. Gen. Physiol..

[69] Hulme J.T., Westenbroek R.E., Scheuer T., Catterall W.A. (2006). Phosphorylation of serine 1928 in the distal C-terminal domain of cardiac cav1.2 channels during β1-adrenergic regulation. Proc. Natl. Acad. Sci. USA.

[70] Appert-Collin A., Cotecchia S., Nenniger-Tosato M., Pedrazzini T., Diviani D. (2007). The A-kinase anchoring protein (AKAP)-lbc-signaling complex mediates alpha1 adrenergic receptor-induced cardiomyocyte hypertrophy. Proc. Natl Acad Sci USA.

[71] Dodge-Kafka K.L., Soughayer J., Pare G.C., Carlisle Michel J.J., Langeberg L.K., Kapiloff M.S., Scott J.D. (2005). The protein kinase a anchoring protein mAKAP coordinates two integrated cAMP effector pathways. Nature.

[72] Fan G., Shumay E., Wang H., Malbon C.C. (2001). The scaffold protein gravin (cAMP-dependent protein kinase-anchoring protein 250) binds the β2-adrenergic receptor via the receptor cytoplasmic Arg-329 to Leu-413 domain and provides a mobile scaffold during desensitization. J. Biol. Chem..

[73] Gardner L.A., Tavalin S.J., Goehring A.S., Scott J.D., Bahouth S.W. (2006). AKAP79-mediated targeting of the cyclic AMP-dependent protein kinase to the β1-adrenergic receptor promotes recycling and functional resensitization of the receptor. J. Biol. Chem..

[74] Chiu J.J., Chien S. (2011). Effects of disturbed flow on vascular endothelium: Pathophysiological basis and clinical perspectives. Physiol. Rev..

[75] Mehta D., Malik A.B. (2006). Signaling mechanisms regulating endothelial permeability. Physiol. Rev..

[76] Cinel I., Dellinger R.P. (2007). Advances in pathogenesis and management of sepsis. Curr. Opin. Infect. Dis..

[77] Choi Y.K., Kim J.H., Kim W.J., Lee H.Y., Park J.A., Lee S.W., Yoon D.K., Kim H.H., Chung H., Yu Y.S. (2007). AKAP12 regulates human blood-retinal barrier formation by downregulation of hypoxia-inducible factor-1α. J. Neurosci..

[78] Schlegel N., Waschke J. (2014). cAMP with other signaling cues converges on rac1 to stabilize the endothelial barrier—A signaling pathway compromised in inflammation. Cell Tissue Res..

[79] Sehrawat S., Ernandez T., Cullere X., Takahashi M., Ono Y., Komarova Y., Mayadas T.N. (2011). AKAP9 regulation of microtubule dynamics promotes epac1-induced endothelial barrier properties. Blood.

[80] Navedo M.F., Nieves-Cintron M., Amberg G.C., Yuan C., Votaw V.S., Lederer W.J., McKnight G.S., Santana L.F. (2008). AKAP150 is required for stuttering persistent Ca^2+^ sparklets and angiotensin ii-induced hypertension. Circ. Res..

[81] Navedo M.F., Cheng E.P., Yuan C., Votaw S., Molkentin J.D., Scott J.D., Santana L.F. (2010). Increased coupled gating of l-type Ca^2+^ channels during hypertension and timothy syndrome. Circ. Res..

[82] Dixon R.E., Cheng E.P., Mercado J.L., Santana L.F. (2012). L-type Ca^2+^ channel function during timothy syndrome. Trends Cardiovasc. Med..

[83] Earley S., Heppner T.J., Nelson M.T., Brayden J.E. (2005). Trpv4 forms a novel Ca^2+^ signaling complex with ryanodine receptors and bkca channels. Circ. Res..

[84] Earley S., Pauyo T., Drapp R., Tavares M.J., Liedtke W., Brayden J.E. (2009). Trpv4-dependent dilation of peripheral resistance arteries influences arterial pressure. Am. J. Physiol. Heart Circ. Physiol..

[85] Bers D.M. (2002). Cardiac excitation-contraction coupling. Nature.

[86] Szentesi P., Pignier C., Egger M., Kranias E.G., Niggli E. (2004). Sarcoplasmic reticulum Ca^2+^ refilling controls recovery from Ca^2+^-induced Ca^2+^ release refractoriness in heart muscle. Circ. Res..

[87] Kranias E.G., Hajjar R.J. (2012). Modulation of cardiac contractility by the phospholamban/serca2a regulatome. Circ. Res..

[88] Bünemann M., Gerhardstein B.L., Gao T., Hosey M.M. (1999). Functional regulation of l-type calcium channels via protein kinase A-mediated phosphorylation of the β2 subunit. J. Biol. Chem..

[89] Nichols C.B., Rossow C.F., Navedo M.F., Westenbroek R.E., Catterall W.A., Santana L.F., McKnight G.S. (2010). Sympathetic stimulation of adult cardiomyocytes requires association of AKAP5 with a subpopulation of l-type calcium channels. Circ. Res..

[90] Schulze D.H., Muqhal M., Lederer W.J., Ruknudin A.M. (2003). Sodium/calcium exchanger (ncx1) macromolecular complex. J. Biol. Chem..

[91] Ruknudin A., He S., Lederer W.J., Schulze D.H. (2000). Functional differences between cardiac and renal isoforms of the rat Na^+^-Ca^2+^ exchanger ncx1 expressed in xenopus oocytes. J. Physiol..

[92] Johnson K.R., Nicodemus-Johnson J., Carnegie G.K., Danziger R.S. (2012). Molecular evolution of A-kinase anchoring protein (AKAP)-7: Implications in comparative PKA compartmentalization. BMC Evol. Biol..

[93] Nerbonne J.M., Kass R.S. (2005). Molecular physiology of cardiac repolarization. Physiol. Rev..

[94] Lu J.T., Kass R.S. (2010). Recent progress in congenital long qt syndrome. Curr. Opin. Cardiol..

[95] Kammerer S., Burns-Hamuro L.L., Ma Y., Hamon S.C., Canaves J.M., Shi M.M., Nelson M.R., Sing C.F., Cantor C.R., Taylor S.S. (2003). Amino acid variant in the kinase binding domain of dual-specific a kinase-anchoring protein 2: A disease susceptibility polymorphism. Proc. Natl. Acad. Sci. USA.

[96] Tingley W.G., Pawlikowska L., Zaroff J.G., Kim T., Nguyen T., Young S.G., Vranizan K., Kwok P.Y., Whooley M.A., Conklin B.R. (2007). Gene-trapped mouse embryonic stem cell-derived cardiac myocytes and human genetics implicate AKAP10 in heart rhythm regulation. Proc. Natl. Acad. Sci. USA.

[97] Łoniewska B., Kaczmarczyk M., Clark J.S., Gorący I., Horodnicka-Józwa A., Ciechanowicz A. (2015). Association of functional genetic variants of A-kinase anchoring protein 10 with qt interval length in full-term polish newborns. Arch. Med. Sci..

[98] Frey N., Olson E.N. (2003). Cardiac hypertrophy: The good, the bad, and the ugly. Annu. Rev. Physiol..

[99] Diviani D., Soderling J., Scott J.D. (2001). AKAP-lbc anchors protein kinase A and nucleates Gα_12_-selective rho-mediated stress fiber formation. J. Biol. Chem..

[100] Klussmann E., Edemir B., Pepperle B., Tamma G., Henn V., Klauschenz E., Hundsrucker C., Maric K., Rosenthal W. (2001). Ht31: The first protein kinase A anchoring protein to integrate protein kinase A and rho signaling. FEBS Lett..

[101] Abdul Azeez K.R., Knapp S., Fernandes J.M., Klussmann E., Elkins J.M. (2014). The crystal structure of the rhoa-AKAP-lbc dh-ph domain complex. Biochem. J..

[102] Schrade K., Tröger J., Eldahshan A., Zühlke K., Abdul Azeez K.R., Elkins J.M., Neuenschwander M., Oder A., Elkewedi M., Jaksch S. (2018). An AKAP-lbc-rhoa interaction inhibitor promotes the translocation of aquaporin-2 to the plasma membrane of renal collecting duct principal cells. PLoS One.

[103] Mayers C.M., Wadell J., McLean K., Venere M., Malik M., Shibata T., Driggers P.H., Kino T., Guo X.C., Koide H. (2010). The rho guanine nucleotide exchange factor AKAP13 (brx) is essential for cardiac development in mice. J. Biol. Chem..

[104] Diviani D., Abuin L., Cotecchia S., Pansier L. (2004). Anchoring of both PKA and 14-3-3 inhibits the rho-gef activity of the AKAP-lbc signaling complex. EMBO J..

[105] Carnegie G.K., Smith F.D., McConnachie G., Langeberg L.K., Scott J.D. (2004). AKAP-lbc nucleates a protein kinase D activation scaffold. Mol. Cell.

[106] Carnegie G.K., Soughayer J., Smith F.D., Pedroja B.S., Zhang F., Diviani D., Bristow M.R., Kunkel M.T., Newton A.C., Langeberg L.K. (2008). AKAP-lbc mobilizes a cardiac hypertrophy signaling pathway. Mol. Cell.

[107] Pare G.C., Easlick J.L., Mislow J.M., McNally E.M., Kapiloff M.S. (2005). Nesprin-1alpha contributes to the targeting of mAKAP to the cardiac myocyte nuclear envelope. Exp. Cell Res..

[108] Zhang L., Malik S., Kelley G.G., Kapiloff M.S., Smrcka A.V. (2011). Phospholipase c epsilon scaffolds to muscle-specific a kinase anchoring protein (mAKAPβ) and integrates multiple hypertrophic stimuli in cardiac myocytes. J. Biol. Chem..

[109] Dodge-Kafka K.L., Bauman A., Mayer N., Henson E., Heredia L., Ahn J., McAvoy T., Nairn A.C., Kapiloff M.S. (2010). Camp-stimulated protein phosphatase 2a activity associated with muscle a kinase-anchoring protein (mAKAP) signaling complexes inhibits the phosphorylation and activity of the cAMP-specific phosphodiesterase pde4d3. J. Biol. Chem..

[110] Wong W., Goehring A.S., Kapiloff M.S., Langeberg L.K., Scott J.D. (2008). MAKAP compartmentalizes oxygen-dependent control of hif-1α. Sci. Signal..

[111] Abrenica B., AlShaaban M., Czubryt M.P. (2009). The a-kinase anchor protein AKAP121 is a negative regulator of cardiomyocyte hypertrophy. J. Mol. Cell. Cardiol.

[112] Means C.K., Xiao C.Y., Li Z., Zhang T., Omens J.H., Ishii I., Chun J., Brown J.H. (2007). Sphingosine 1-phosphate s1p2 and s1p3 receptor-mediated akt activation protects against in vivo myocardial ischemia-reperfusion injury. Am. J. Physiol. Heart Circ. Physiol..

[113] Schiattarella G.G., Cattaneo F., Pironti G., Magliulo F., Carotenuto G., Pirozzi M., Polishchuk R., Borzacchiello D., Paolillo R., Oliveti M. (2016). AKAP1 deficiency promotes mitochondrial aberrations and exacerbates cardiac injury following permanent coronary ligation via enhanced mitophagy and apoptosis. PLoS One.

[114] Lacaná E., Maceyka M., Milstien S., Spiegel S. (2002). Cloning and characterization of a protein kinase a anchoring protein (AKAP)-related protein that interacts with and regulates sphingosine kinase 1 activity. J. Biol. Chem..

[115] Vasudevan N.T., Mohan M.L., Goswami S.K., Naga Prasad S.V. (2011). Regulation of β-adrenergic receptor function: An emphasis on receptor resensitization. Cell Cycle.

[116] Lohse M.J., Engelhardt S., Eschenhagen T. (2003). What is the role of β-adrenergic signaling in heart failure?. Circ. Res..

[117] Gold M.G., Gonen T., Scott J.D. (2013). Local cAMP signaling in disease at a glance. J. Cell Sci..

[118] Berisha F., Nikolaev V.O. (2017). Cyclic nucleotide imaging and cardiovascular disease. Pharmacol. Ther..

[119] Froese A., Nikolaev V.O. (2015). Imaging alterations of cardiomyocyte cAMP microdomains in disease. Front. Pharmacol..

[120] Pavlaki N., Nikolaev V.O. (2018). Imaging of PDE2- and PDE3-mediated cGMP-to-cAMP cross-talk in cardiomyocytes. J. Cardiovasc. Dev. Dis..

[121] Musheshe N., Schmidt M., Zaccolo M. (2017). cAMP: From long-range second messenger to nanodomain signalling. Trends Pharmacol. Sci..

[122] Thestrup T., Litzlbauer J., Bartholomäus I., Mues M., Russo L., Dana H., Kovalchuk Y., Liang Y., Kalamakis G., Laukat Y. (2014). Optimized ratiometric calcium sensors for functional in vivo imaging of neurons and t lymphocytes. Nat. Methods.

[123] Sprenger J.U., Nikolaev V.O. (2013). Biophysical techniques for detection of cAMP and cGMP in living cells. Int J. Mol. Sci..

[124] Perera R.K., Sprenger J.U., Steinbrecher J.H., Hübscher D., Lehnart S.E., Abesser M., Schuh K., El-Armouche A., Nikolaev V.O. (2015). Microdomain switch of cGMP-regulated phosphodiesterases leads to anp-induced augmentation of β-adrenoceptor-stimulated contractility in early cardiac hypertrophy. Circ. Res..

[125] Sprenger J.U., Perera R.K., Steinbrecher J.H., Lehnart S.E., Maier L.S., Hasenfuss G., Nikolaev V.O. (2015). In vivo model with targeted cAMP biosensor reveals changes in receptor-microdomain communication in cardiac disease. Nat. Commun..

[126] Jungen C., Scherschel K., Eickholt C., Kuklik P., Klatt N., Bork N., Salzbrunn T., Alken F., Angendohr S., Klene C. (2017). Disruption of cardiac cholinergic neurons enhances susceptibility to ventricular arrhythmias. Nat. Commun..

[127] Sanchez-Alonso J.L., Bhargava A., O’Hara T., Glukhov A.V., Schobesberger S., Bhogal N., Sikkel M.B., Mansfield C., Korchev Y.E., Lyon A.R. (2016). Microdomain-specific modulation of l-type calcium channels leads to triggered ventricular arrhythmia in heart failure. Circ. Res..

[128] Calebiro D., Rieken F., Wagner J., Sungkaworn T., Zabel U., Borzi A., Cocucci E., Zürn A., Lohse M.J. (2013). Single-molecule analysis of fluorescently labeled g-protein-coupled receptors reveals complexes with distinct dynamics and organization. Proc. Natl Acad Sci USA.

[129] Miragoli M., Moshkov A., Novak P., Shevchuk A., Nikolaev V.O., El-Hamamsy I., Potter C.M., Wright P., Kadir S.H., Lyon A.R. (2011). Scanning ion conductance microscopy: A convergent high-resolution technology for multi-parametric analysis of living cardiovascular cells. J. R Soc. Interface.

[130] Nikolaev V.O., Moshkov A., Lyon A.R., Miragoli M., Novak P., Paur H., Lohse M.J., Korchev Y.E., Harding S.E., Gorelik J. (2010). β2-adrenergic receptor redistribution in heart failure changes cAMP compartmentation. Science.

[131] Conti M., Beavo J. (2007). Biochemistry and physiology of cyclic nucleotide phosphodiesterases: Essential components in cyclic nucleotide signaling. Annu. Rev. Biochem..

[132] Maurice D.H., Palmer D., Tilley D.G., Dunkerley H.A., Netherton S.J., Raymond D.R., Elbatarny H.S., Jimmo S.L. (2003). Cyclic nucleotide phosphodiesterase activity, expression, and targeting in cells of the cardiovascular system. Mol. Pharmacol..

[133] Keravis T., Lugnier C. (2010). Cyclic nucleotide phosphodiesterases (PDE) and peptide motifs. Curr. Pharm. Des..

[134] Anant J.S., Ong O.C., Xie H.Y., Clarke S., O’Brien P.J., Fung B.K. (1992). In vivo differential prenylation of retinal cyclic gmp phosphodiesterase catalytic subunits. J. Biol. Chem..

[135] Baillie G.S., MacKenzie S.J., McPhee I., Houslay M.D. (2000). Sub-family selective actions in the ability of erk2 map kinase to phosphorylate and regulate the activity of PDE4 cyclic AMP-specific phosphodiesterases. Br. J. Pharmacol..

[136] Omori K., Kotera J. (2007). Overview of pdes and their regulation. Circ. Res..

[137] Francis S.H., Blount M.A., Corbin J.D. (2011). Mammalian cyclic nucleotide phosphodiesterases: Molecular mechanisms and physiological functions. Physiol. Rev..

[138] Ke H., Wang H. (2007). Crystal structures of phosphodiesterases and implications on substrate specificity and inhibitor selectivity. Curr. Top. Med. Chem..

[139] Wechsler J., Choi Y.H., Krall J., Ahmad F., Manganiello V.C., Movsesian M.A. (2002). Isoforms of cyclic nucleotide phosphodiesterase PDE3a in cardiac myocytes. J. Biol. Chem..

[140] Senzaki H., Smith C.J., Juang G.J., Isoda T., Mayer S.P., Ohler A., Paolocci N., Tomaselli G.F., Hare J.M., Kass D.A. (2001). Cardiac phosphodiesterase 5 (cGMP-specific) modulates β-adrenergic signaling in vivo and is down-regulated in heart failure. FASEB J..

[141] Rosman G.J., Martins T.J., Sonnenburg W.K., Beavo J.A., Ferguson K., Loughney K. (1997). Isolation and characterization of human cdnas encoding a cGMP-stimulated 3′,5′-cyclic nucleotide phosphodiesterase. Gene.

[142] Zhang H., Liu X.H., Zhang K., Chen C.K., Frederick J.M., Prestwich G.D., Baehr W. (2004). Photoreceptor cgmp phosphodiesterase delta subunit (pdedelta) functions as a prenyl-binding protein. J. Biol. Chem..

[143] Han P., Sonati P., Rubin C., Michaeli T. (2006). Pde7a1, a cAMP-specific phosphodiesterase, inhibits cAMP-dependent protein kinase by a direct interaction with c. J. Biol. Chem..

[144] Wang P., Wu P., Egan R.W., Billah M.M. (2003). Identification and characterization of a new human type 9 cGMP-specific phosphodiesterase splice variant (PDE9a5). Differential tissue distribution and subcellular localization of PDE9a variants. Gene.

[145] Sayner S., Stevens T. (2006). Soluble adenylate cyclase reveals the significance of compartmentalized cAMP on endothelial cell barrier function. Biochem. Soc. Trans..

[146] Creighton J., Zhu B., Alexeyev M., Stevens T. (2008). Spectrin-anchored phosphodiesterase 4d4 restricts cAMP from disrupting microtubules and inducing endothelial cell gap formation. J. Cell. Sci..

[147] Mongillo M., Tocchetti C.G., Terrin A., Lissandron V., Cheung Y.F., Dostmann W.R., Pozzan T., Kass D.A., Paolocci N., Houslay M.D. (2006). Compartmentalized phosphodiesterase-2 activity blunts β-adrenergic cardiac inotropy via an no/cGMP-dependent pathway. Circ. Res..

[148] Beca S., Helli P.B., Simpson J.A., Zhao D., Farman G.P., Jones P., Tian X., Wilson L.S., Ahmad F., Chen S.R.W. (2011). Phosphodiesterase 4d regulates baseline sarcoplasmic reticulum Ca^2+^ release and cardiac contractility, independently of l-type Ca^2+^ current. Circ. Res..

[149] Borlaug B.A., Melenovsky V., Marhin T., Fitzgerald P., Kass D.A. (2005). Sildenafil inhibits β-adrenergic-stimulated cardiac contractility in humans. Circulation.

[150] Patrucco E., Albergine M.S., Santana L.F., Beavo J.A. (2010). Phosphodiesterase 8a (PDE8a) regulates excitation-contraction coupling in ventricular myocytes. J. Mol. Cell. Cardiol..

[151] Galindo-Tovar A., Kaumann A.J. (2008). Phosphodiesterase-4 blunts inotropism and arrhythmias but not sinoatrial tachycardia of (−)-adrenaline mediated through mouse cardiac β(1)-adrenoceptors. Br. J. Pharmacol..

[152] Ding B., Abe J., Wei H., Xu H., Che W., Aizawa T., Liu W., Molina C.A., Sadoshima J., Blaxall B.C. (2005). A positive feedback loop of phosphodiesterase 3 (PDE3) and inducible cAMP early repressor (icer) leads to cardiomyocyte apoptosis. Proc. Natl. Acad. Sci. USA.

[153] Leroy J., Richter W., Mika D., Castro L.R., Abi-Gerges A., Xie M., Scheitrum C., Lefebvre F., Schittl J., Mateo P. (2011). Phosphodiesterase 4b in the cardiac l-type Ca^2+^ channel complex regulates Ca^2+^ current and protects against ventricular arrhythmias in mice. J. Clin. Invest..

[154] Lehnart S.E., Wehrens X.H., Reiken S., Warrier S., Belevych A.E., Harvey R.D., Richter W., Jin S.L., Conti M., Marks A.R. (2005). Phosphodiesterase 4d deficiency in the ryanodine-receptor complex promotes heart failure and arrhythmias. Cell.

[155] Pokreisz P., Vandenwijngaert S., Bito V., Van den Bergh A., Lenaerts I., Busch C., Marsboom G., Gheysens O., Vermeersch P., Biesmans L. (2009). Ventricular phosphodiesterase-5 expression is increased in patients with advanced heart failure and contributes to adverse ventricular remodeling after myocardial infarction in mice. Circulation.

[156] Lee D.I., Zhu G., Sasaki T., Cho G.S., Hamdani N., Holewinski R., Jo S.H., Danner T., Zhang M., Rainer P.P. (2015). Phosphodiesterase 9a controls nitric-oxide-independent cGMP and hypertrophic heart disease. Nature.

[157] Hambleton R., Krall J., Tikishvili E., Honeggar M., Ahmad F., Manganiello V.C., Movsesian M.A. (2005). Isoforms of cyclic nucleotide phosphodiesterase pde3 and their contribution to cAMP hydrolytic activity in subcellular fractions of human myocardium. J. Biol. Chem..

[158] Ahmad F., Murata T., Shimizu K., Degerman E., Maurice D., Manganiello V. (2015). Cyclic nucleotide phosphodiesterases: Important signaling modulators and therapeutic targets. Oral Dis..

[159] Maass P.G., Aydin A., Luft F.C., Schächterle C., Weise A., Stricker S., Lindschau C., Vaegler M., Qadri F., Toka H.R. (2015). PDE3a mutations cause autosomal dominant hypertension with brachydactyly. Nat. Genet..

[160] Hattenbach L.O., Toka H.R., Toka O., Schuster H., Luft F.C. (1998). Absence of hypertensive retinopathy in a turkish kindred with autosomal dominant hypertension and brachydactyly. Br. J. Ophthalmol..

[161] Toka O., Tank J., Schächterle C., Aydin A., Maass P.G., Elitok S., Bartels-Klein E., Hollfinger I., Lindschau C., Mai K. (2015). Clinical effects of phosphodiesterase 3a mutations in inherited hypertension with brachydactyly. Hypertension.

[162] Movsesian M. (2016). Novel approaches to targeting PDE3 in cardiovascular disease. Pharmacol. Ther..

[163] Movsesian M., Ahmad F., Hirsch E. (2018). Functions of PDE3 isoforms in cardiac muscle. J. Cardiovasc. Dev. Dis.

[164] Francis S.H., Conti M., Houslay M.D. (2011). Handbook of Experimental Pharmacology 204. Phosphodiesterases as Drug Targets.

[165] Knight W., Yan C. (2013). Therapeutic potential of pde modulation in treating heart disease. Future Med. Chem..

[166] Liu Y., Shakur Y., Kambayashi J. (2011). Phosphodiesterases as targets for intermittent claudication. Handb Exp. Pharmacol..

[167] Rogers K.C., Oliphant C.S., Finks S.W. (2015). Clinical efficacy and safety of cilostazol: A critical review of the literature. Drugs.

[168] Hiatt W.R., Money S.R., Brass E.P. (2008). Long-term safety of cilostazol in patients with peripheral artery disease: The castle study (cilostazol: A study in long-term effects). J. Vasc. Surg..

[169] Takigawa T., Matsumaru Y., Hayakawa M., Nemoto S., Matsumura A. (2010). Cilostazol reduces restenosis after carotid artery stenting. J. Vasc. Surg..

[170] Cone J., Wang S., Tandon N., Fong M., Sun B., Sakurai K., Yoshitake M., Kambayashi J., Liu Y. (1999). Comparison of the effects of cilostazol and milrinone on intracellular cAMP levels and cellular function in platelets and cardiac cells. J. Cardiovasc. Pharmacol..

[171] Faxon D.P., Creager M.A., Smith S.C., Pasternak R.C., Olin J.W., Bettmann M.A., Criqui M.H., Milani R.V., Loscalzo J., Kaufman J.A. (2004). Atherosclerotic vascular disease conference: Executive summary: Atherosclerotic vascular disease conference proceeding for healthcare professionals from a special writing group of the american heart association. Circulation.

[172] Gresele P., Momi S., Falcinelli E. (2011). Anti-platelet therapy: Phosphodiesterase inhibitors. Br. J. Clin. Pharmacol..

[173] Sahin M., Alizade E., Pala S., Alici G., Ozkan B., Akgun T., Emiroglu Y., Demir S., Yazicioglu M.V., Turkmen M.M. (2013). The effect of cilostazol on right heart function and pulmonary pressure. Cardiovasc. Ther..

[174] Inoue Y., Toga K., Sudo T., Tachibana K., Tochizawa S., Kimura Y., Yoshida Y., Hidaka H. (2000). Suppression of arterial intimal hyperplasia by cilostamide, a cyclic nucleotide phosphodiesterase 3 inhibitor, in a rat balloon double-injury model. Br. J. Pharmacol..

[175] Overgaard C.B., Dzavík V. (2008). Inotropes and vasopressors: Review of physiology and clinical use in cardiovascular disease. Circulation.

[176] Tariq S., Aronow W.S. (2015). Use of inotropic agents in treatment of systolic heart failure. Int J. Mol. Sci..

[177] Movsesian M. (2015). New pharmacologic interventions to increase cardiac contractility: Challenges and opportunities. Curr. Opin. Cardiol..

[178] Klussmann E. (2016). Protein-protein interactions of PDE4 family members—Functions, interactions and therapeutic value. Cell Signal..

[179] Sims C.R., Singh S.P., Mu S., Gokden N., Zakaria D., Nguyen T.C., Mayeux P.R. (2017). Rolipram improves outcome in a rat model of infant sepsis-induced cardiorenal syndrome. Front. Pharmacol..

[180] Lin Y.C., Samardzic H., Adamson R.H., Renkin E.M., Clark J.F., Reed R.K., Curry F.R. (2011). Phosphodiesterase 4 inhibition attenuates atrial natriuretic peptide-induced vascular hyperpermeability and loss of plasma volume. J. Physiol..

[181] Richter W., Xie M., Scheitrum C., Krall J., Movsesian M.A., Conti M. (2011). Conserved expression and functions of PDE4 in rodent and human heart. Basic Res. Cardiol..

[182] Rabe K.F. (2011). Update on roflumilast, a phosphodiesterase 4 inhibitor for the treatment of chronic obstructive pulmonary disease. Br. J. Pharmacol..

[183] Tenor H., Hatzelmann A., Beume R., Lahu G., Zech K., Bethke T.D. (2011). Pharmacology, clinical efficacy, and tolerability of phosphodiesterase-4 inhibitors: Impact of human pharmacokinetics. Handb Exp. Pharmacol..

[184] Sakkas L.I., Mavropoulos A., Bogdanos D.P. (2017). Phosphodiesterase 4 inhibitors in immune-mediated diseases: Mode of action, clinical applications, current and future perspectives. Curr. Med. Chem..

[185] Keating G.M. (2017). Apremilast: A review in psoriasis and psoriatic arthritis. Drugs.

[186] Nagendran J., Archer S.L., Soliman D., Gurtu V., Moudgil R., Haromy A., St Aubin C., Webster L., Rebeyka I.M., Ross D.B. (2007). Phosphodiesterase type 5 is highly expressed in the hypertrophied human right ventricle, and acute inhibition of phosphodiesterase type 5 improves contractility. Circulation.

[187] Gong W., Yan M., Chen J., Chaugai S., Chen C., Wang D. (2014). Chronic inhibition of cyclic guanosine monophosphate-specific phosphodiesterase 5 prevented cardiac fibrosis through inhibition of transforming growth factor β-induced smad signaling. Front. Med..

[188] Guazzi M., van Heerebeek L., Paulus W.J. (2017). Phosphodiesterase-5 inhibition in heart failure with preserved ejection fraction: Trading therapy for prevention. Eur J. Heart Fail..

[189] Katz S.D., Balidemaj K., Homma S., Wu H., Wang J., Maybaum S. (2000). Acute type 5 phosphodiesterase inhibition with sildenafil enhances flow-mediated vasodilation in patients with chronic heart failure. J. Am. Coll. Cardiol..

[190] Kukreja R.C., Salloum F.N., Das A., Koka S., Ockaili R.A., Xi L. (2011). Emerging new uses of phosphodiesterase-5 inhibitors in cardiovascular diseases. Exp. Clin. Cardiol..

[191] Pofi R., Gianfrilli D., Badagliacca R., Di Dato C., Venneri M.A., Giannetta E. (2016). Everything you ever wanted to know about phosphodiesterase 5 inhibitors and the heart (but never dared ask): How do they work?. J. Endocrinol. Invest..

[192] Sonnenburg W.K., Seger D., Beavo J.A. (1993). Molecular cloning of a cdna encoding the “61-kda” Calmodulin-stimulated cyclic nucleotide phosphodiesterase. Tissue-specific expression of structurally related isoforms. J. Biol. Chem..

[193] Goraya T.A., Cooper D.M. (2005). Ca^2+^-calmodulin-dependent phosphodiesterase (pde1): Current perspectives. Cell Signal..

[194] Saeki T., Adachi H., Takase Y., Yoshitake S., Souda S., Saito I. (1995). A selective type v phosphodiesterase inhibitor, e4021, dilates porcine large coronary artery. J. Pharmacol. Exp. Ther..

[195] Nagel D.J., Aizawa T., Jeon K.I., Liu W., Mohan A., Wei H., Miano J.M., Florio V.A., Gao P., Korshunov V.A. (2006). Role of nuclear Ca^2+^/calmodulin-stimulated phosphodiesterase 1a in vascular smooth muscle cell growth and survival. Circ. Res..

[196] Schermuly R.T., Pullamsetti S.S., Kwapiszewska G., Dumitrascu R., Tian X., Weissmann N., Ghofrani H.A., Kaulen C., Dunkern T., Schudt C. (2007). Phosphodiesterase 1 upregulation in pulmonary arterial hypertension: Target for reverse-remodeling therapy. Circulation.

[197] Fischmeister R., Castro L., Abi-Gerges A., Rochais F., Vandecasteele G. (2005). Species- and tissue-dependent effects of no and cyclic gmp on cardiac ion channels. Comp. Biochem. Physiol. A Mol. Integr. Physiol..

[198] Mehel H., Emons J., Vettel C., Wittköpper K., Seppelt D., Dewenter M., Lutz S., Sossalla S., Maier L.S., Lechêne P. (2013). Phosphodiesterase-2 is up-regulated in human failing hearts and blunts β-adrenergic responses in cardiomyocytes. J. Am. Coll. Cardiol..

[199] Vettel C., Lindner M., Dewenter M., Lorenz K., Schanbacher C., Riedel M., Lämmle S., Meinecke S., Mason F.E., Sossalla S. (2017). Phosphodiesterase 2 protects against catecholamine-induced arrhythmia and preserves contractile function after myocardial infarction. Circ. Res..

[200] Rentero C., Monfort A., Puigdomènech P. (2003). Identification and distribution of different mrna variants produced by differential splicing in the human phosphodiesterase 9a gene. Biochem. Biophys. Res. Commun..

[201] Christian F., Szaszák M., Friedl S., Drewianka S., Lorenz D., Goncalves A., Furkert J., Vargas C., Schmieder P., Götz F. (2011). Small molecule AKAP-protein kinase a (PKA) interaction disruptors that activate PKA interfere with compartmentalized cAMP signaling in cardiac myocytes. J. Biol. Chem..

[202] Khan A., Munir M., Aiman S., Wadood A., Khan A.U. (2017). The in silico identification of small molecules for protein-protein interaction inhibition in AKAP-lbc-rhoa signaling complex. Comput. Biol. Chem..

[203] Diviani D., Raimondi F., Del Vescovo C.D., Dreyer E., Reggi E., Osman H., Ruggieri L., Gonano C., Cavin S., Box C.L. (2016). Small-molecule protein-protein interaction inhibitor of oncogenic rho signaling. Cell Chem. Biol..

[204] Serrels B., Sandilands E., Serrels A., Baillie G., Houslay M.D., Brunton V.G., Canel M., Machesky L.M., Anderson K.I., Frame M.C. (2010). A complex between fak, rack1, and PDE4d5 controls spreading initiation and cancer cell polarity. Curr. Biol..

